# Recurrent basosquamous carcinoma of the forearm, a case report

**DOI:** 10.1016/j.jpra.2026.05.005

**Published:** 2026-05-09

**Authors:** Daniel Reiser, Arpad Szallasi, Marcus Sagerfors

**Affiliations:** a Department of Orthopedics and Hand Surgery, Faculty of Medicine and Health, Örebro University, SE 70182 Örebro, Sweden; b Department of Pathology, Faculty of Medicine and Health, Örebro University, SE 70182 Örebro, Sweden; c Department of Pathology and Experimental Cancer Research, Semmelweis University, H-1085 Budapest, Hungary

**Keywords:** Basosquamous carcinoma, Skin cancer, Surgery, Forearm, Negative-pressure wound therapy

## Abstract

Basosquamous carcinoma (BSC) is a rare, aggressive skin cancer with histologic features of both basal and squamous cell carcinoma. This report describes an unusual case of rapidly growing and extensive BSC of the forearm in a 52-year-old male, a rare location for this cancer type. Despite surgical excision with near-complete margins and postoperative wound management using negative-pressure wound therapy (NPWT), the tumor recurred within eight weeks. The case raises concerns about the adequacy of margin control, the possible role of intraoperative tumor cell seeding, and the influence of NPWT and subsequent skin grafting on tumor recurrence. Following re-resection and radiotherapy, the patient achieved excellent functional outcomes, regaining good hand use and returning to competitive motorcycle racing. This case highlights key diagnostic, surgical, and postoperative challenges in managing aggressive BSC in an atypical location and suggests the need for further research into the safety of NPWT in oncologic surgery.

## Introduction

Basosquamous carcinoma (BSC) is an uncommon and clinically challenging cutaneous malignancy characterized by histopathological features of both basal cell carcinoma (BCC) and squamous cell carcinoma (SCC). Though often classified as a rare variant of BCC, BSC exhibits a more aggressive biological behavior, with higher recurrence and metastatic potential compared to conventional BCC, warranting clinical management more akin to SCC in many cases.^1^^,^^2^ The incidence of BSC is estimated to range from 1.2% to 2.7% of all nonmelanoma skin cancers, and the tumor most frequently arises in sun-exposed areas, particularly the head and neck. BSCs occurring on the extremities, such as the forearm, are notably rare. BSC typically affects elderly individuals, especially fair-skinned males, and its diagnosis is often delayed due to its nonspecific clinical appearance, which can mimic either BCC or SCC.^3^^,^^4^ The histopathologic diagnosis of BSC requires the identification of both basaloid and squamous differentiation within the same tumor mass, often in continuity through a transition zone. This dual morphology can make initial biopsy-based diagnoses challenging, particularly if the specimen is superficial or non-representative.^5^ Given its rarity, particularly in younger patients and in atypical anatomical locations such as the forearm, cases of BSC warrant reporting to broaden the clinical understanding of its presentation, growth dynamics, and management. We herein describe a unique case of a rapidly growing BSC on the forearm of a young adult male, emphasizing the tumor’s unusual clinical course and discussing its diagnostic and therapeutic implications.

## Case report

A 52-year-old male with no previous medical history noticed a skin lesion a few millimeters in size on his left forearm. He contacted a general practitioner, who initially treated it as an eczema wound. Within a very short time, the lesion grew massively. Five weeks after the patient's initial consultation, he was seen by a hand surgeon. The tumor had now reached roughly the size of a grapefruit. A biopsy indicated BSC and an MRI was taken, indicating no involvement of the median nerve or the radial nerve. The motor and sensory functions of the hand were normal.

**Fig. 1:**
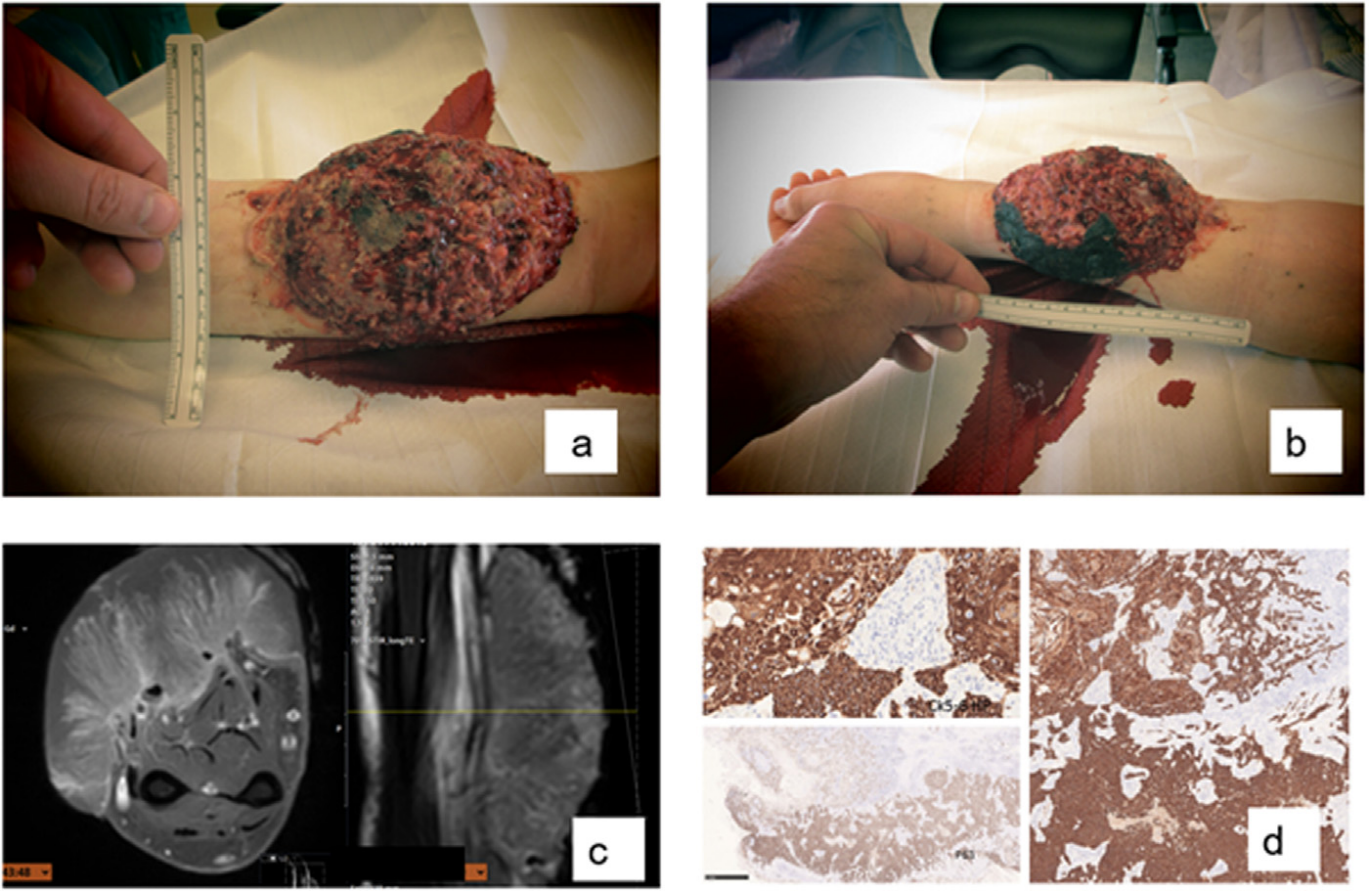
a/b) tumor at first presentation at office; c) pre-operative MRI d) biopsy immunostains from the basosquamous area.

**Fig. 2:**
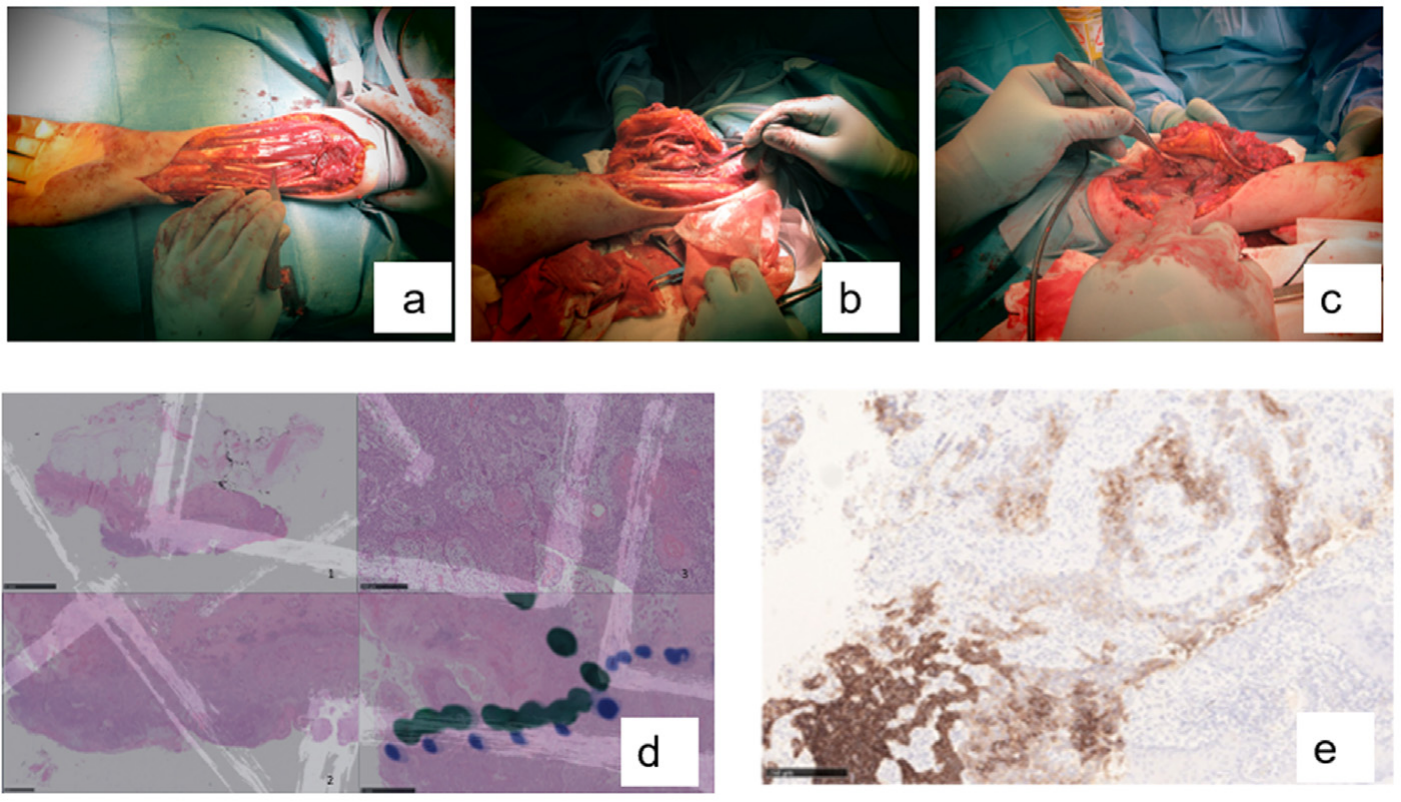
a/b/c) perioperative photos; d) part of the tumor is basal cell carcinoma morphing into squamous cell carcinoma ("basosquamous carcinoma") d) Representative pictures taken at different magnifications. The colored dots show the border between the basal cell and squamous cell components; e) BerEP4 is positive in basal cell carcinoma and negative in squamous cell carcinoma.

Surgery was performed a few days after the initial consultation. The tumor measured 15 × 5.5 cm. It had a foul smell and was disintegrating and necrotic (Fig. 1). Approximately 80% of the superficial digitorum muscle was macroscopically affected. Only the deepest part to the little finger was unaffected. The entire flexor pollicis longus muscle was also affected and was removed, as was the palmaris longus muscle. The tumor also affected the brachioradialis muscle and it was removed. Macroscopically, the entire tumor was removed with a safety margin of 20 mm (mm) from the skin and 10 mm depth. The excision margin of 20 mm recommended by Murgia could not be fully maintained in this case to preserve the median nerve.^6^ The margin was at least 10 mm (Fig. 2 supplement).

**Fig. 3:**
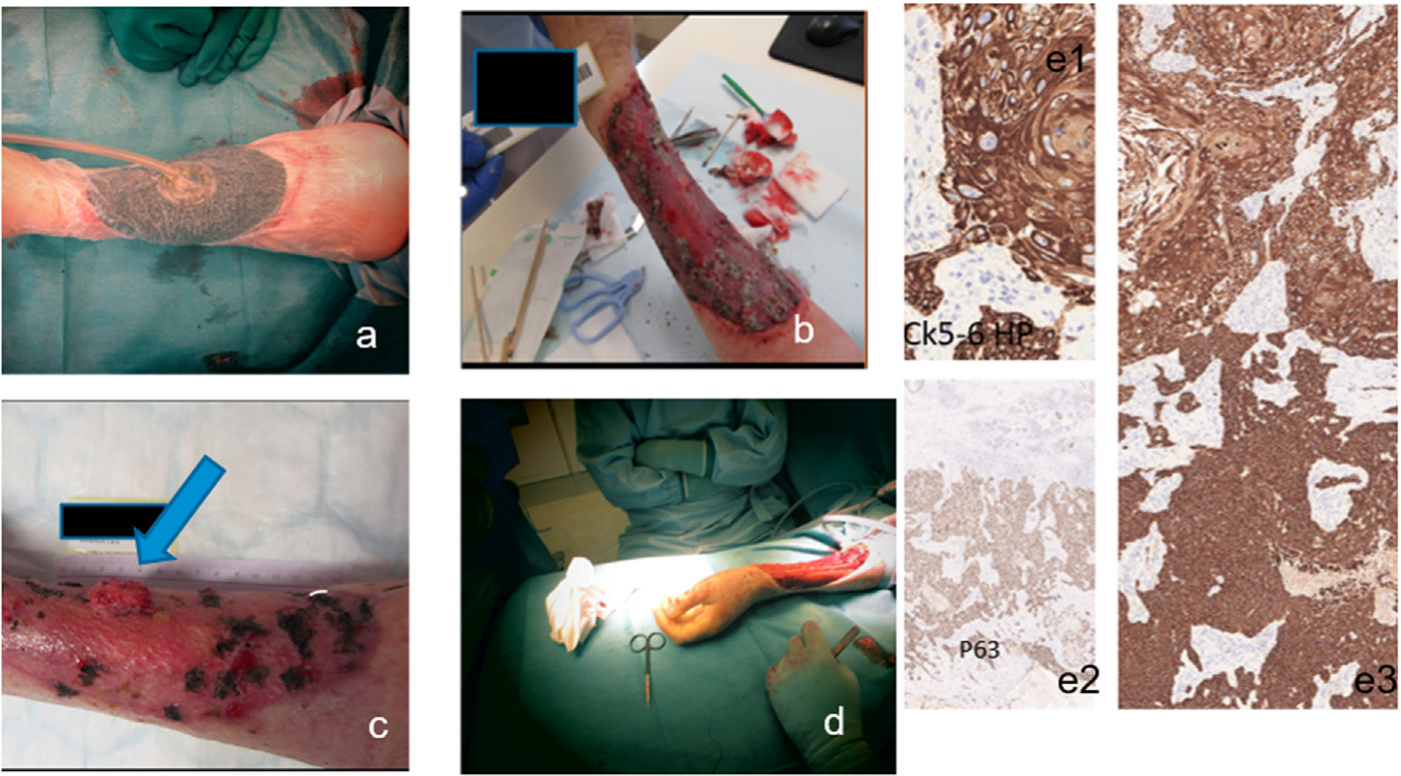
a) NPWT, b) 3 weeks after STSG, c) 6 weeks after STSG, arrow on the recurrent tumor, d) re-operation, e1–3) immunostains from the basosquamous area after recurrence surgery.

Since the pathology report indicated that the resection in a smaller area was without a sufficient marginal, <10 mm, another resection was performed. The subsequent pathology showed a satisfactory marginal area. The wound was treated openly with negative-pressure wound therapy (NPWT) and 19 days after the initial procedure, the wound was covered with a split-thickness skin graft (STSG). Four weeks later, the STSG was healed, but another four weeks later, a tumor recurred (Fig. 3, supplement).

The recurrent tumor was safely removed with a margin of 20 mm including parts of the flexor digitorum superficialis and flexor carpi radialis muscles. Subsequently, radiation treatment with a total of 68 Gy was administered. CT scans were performed every three months from diagnosis. No metastases were detected. One year after the last surgery, the patient had a good hand function. He successfully competes in motorcycle races again and can change gears up to 1500 times during a race. At 2-year follow-up, the patient demonstrated a QuickDASH score of 8, with somewhat reduced range of motion. He was pain free, the grip strength was reduced but he maintained full finger flexion to the distal palmar crease and thumb opposition (Table 1).

**Table 1:** Clinical outcome at last follow-up visit 2 years postoperatively.

Outcome measure	
Flexion°	25
Extension°	15
Pronation°	65
Supination°	5
Radial deviation°	30
Ulnar deviation°	10
Hand grip strength kilograms	17,3
quickDASH score	8
VAS pain at rest	0
VAS pain during activity	0

## Discussion

BSC remains a diagnostically and therapeutically challenging cutaneous malignancy due to its aggressive biological behavior and overlapping histologic features of BCC and SCC.^4^^,^^6^ In this case, the tumor was entirely resected with sufficient margins according to the pathology report, yet a local recurrence occurred within eight weeks. This raises the question of whether residual tumor cells may have been present beyond the histologically assessed margins or possibly introduced into the wound during the surgical manipulation. Murgia recommends a 20 mm marginal excision, which was not fully maintained in this case in order to preserve important nerve structures.^6^ The margin was only 10 mm in some areas.

Another speculative consideration is whether tumor cells might have "seeded" into the wound bed during the procedure.^7^ Although rare, tumor implantation during surgery has been described in other cancers. Moreover, the open wound management with NPWT may have played a role. While widely used to promote granulation and reduce wound size, there is limited literature regarding its application in oncologic settings, particularly after resection of aggressive skin cancers. A theoretical concern is that the subatmospheric pressure could alter local tissue microenvironments or influence cell migration, although direct causality with tumor recurrence has not been proven.^8^ However, a recent meta-analysis found favorable outcomes of NPWT compared to standard surgical dressings for malignancy-resected wounds without an increased risk of malignancy recurrence.^9^

Another consideration involves the subsequent STSG. Radiation therapy was administered after graft healing, delivering 68 Gy in total.^6^ While adjuvant radiation is recommended in high-risk BSC cases, irradiating skin grafts presents unique challenges. Grafted tissues have altered vascularization and healing dynamics, which may affect radiation sensitivity, increase the risk of ulceration or breakdown, and complicate dose distribution. Furthermore, STSGs offer less robust dermal protection compared to full-thickness flaps, potentially influencing recurrence risk in previously infiltrated areas.

Despite these concerns, the long-term functional outcome was favorable. Two years postoperatively, the patient reported excellent use of his hand with a good range of wrist motion and a low quickDASH-score indicating a good hand function, including participation in competitive motorcycle racing. His ability to quickly shift gears demonstrates rehabilitation success.

In conclusion, this case highlights the complexity of managing BSC in rare anatomical locations with substantial soft tissue involvement. It underscores the importance of radical excision, careful consideration of wound management strategies, and potential implications of radiation on reconstructed tissues. Further studies are needed to clarify best practices for NPWT use and adjuvant therapy in BSC cases involving extensive surgical reconstruction.

## Ethical approval

The study was conducted in accordance with the Declaration of Helsinki (as revised in 2013). Oral and written informed consent has been given by the patient. Radiographs and photos are anonymized.

## Funding

None.

## Declaration of competing interest

The authors report there are no competing interests to declare.

## Appendix Supplementary materials

Image, image 1 dummy alt text

Image, image 2 dummy alt text
