# Supplementary figures and images for: Recurrent basosquamous carcinoma of the forearm, a case report
Source: JPRAS Open. 2026 May 9;50:300–4. doi: 10.1016/j.jpra.2026.05.005 (PMC13227292; doi:10.1016/j.jpra.2026.05.005)

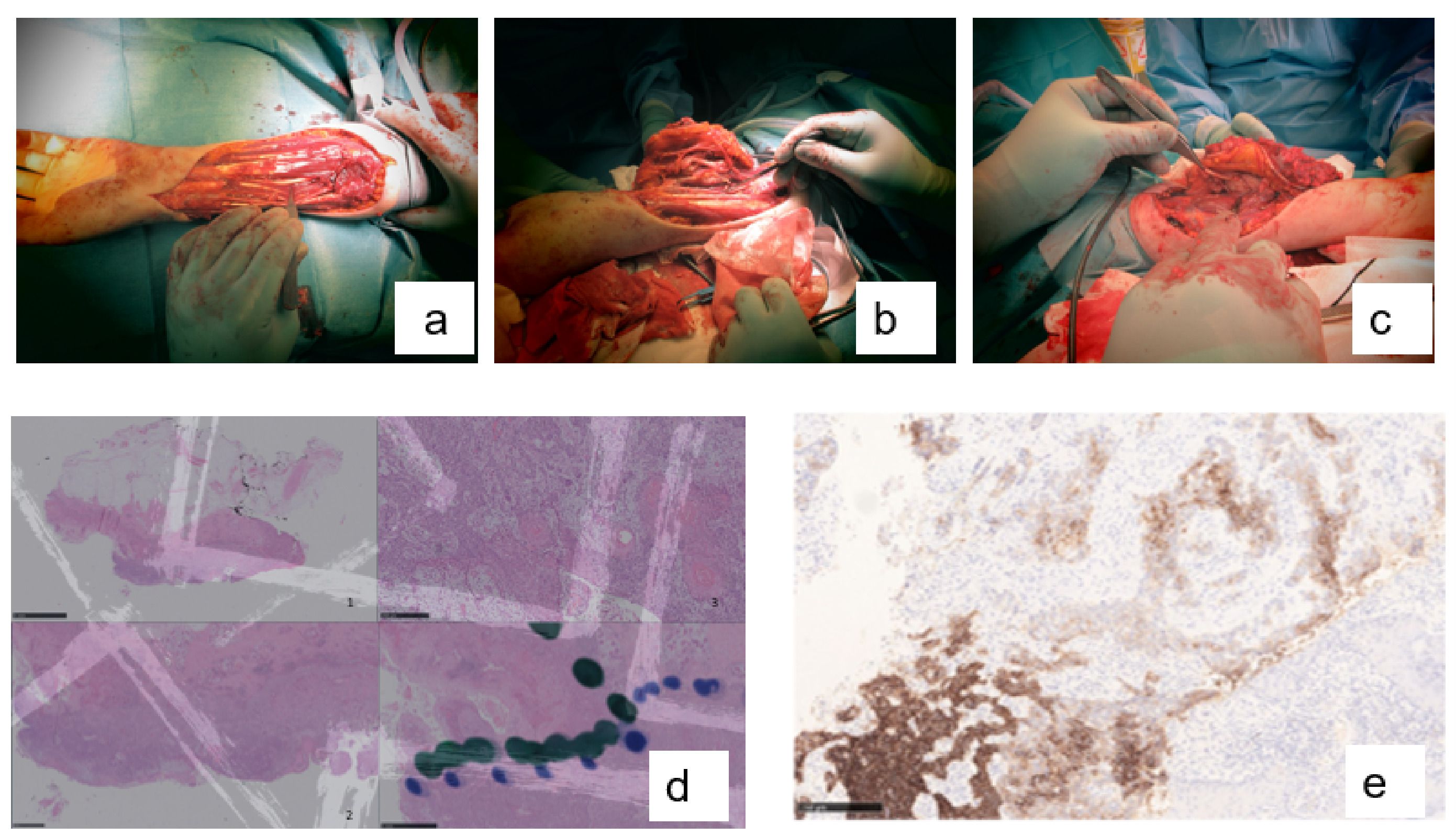

Supplement: Supplementary file 1 [file mmc1.jpg]

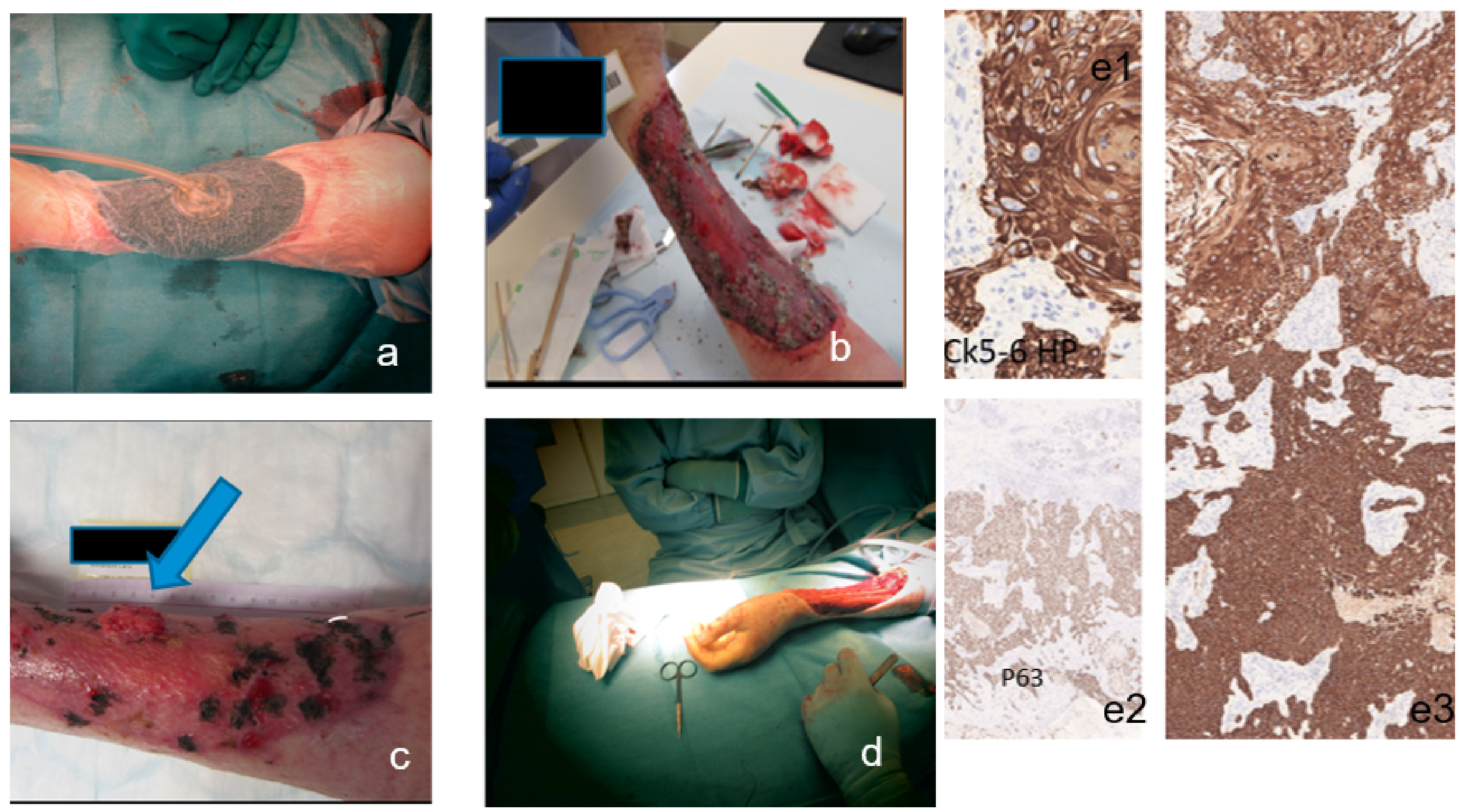

Supplement: Supplementary file 2 [file mmc2.jpg]
